# Epigallocatechin gallate prevents cardiomyocytes from pyroptosis through lncRNA MEG3/TAF15/AIM2 axis in myocardial infarction

**DOI:** 10.1186/s13020-023-00856-z

**Published:** 2023-12-06

**Authors:** Chaoshi Qin, Tingting Wang, Ni Qian, Jing Liu, Rong Xi, Qing Zou, Hui Liu, Xiaolin Niu

**Affiliations:** Department of Cardiology, Tangdu Hospital, Air Force Medical University, No. 569 Xinsi Road, Baqiao District, Xi’an, 710038 Shaanxi Province China

**Keywords:** Pyroptosis, EGCG, LncRNA MEG3, AIM2, TAF15, Myocardial infarction

## Abstract

**Background:**

( −)-Epigallocatechin-3-gallate (EGCG), a bioactive polyphenol isolated from green tea, has recently garnered attention for its potential protective role against acute myocardial infarction (MI) via inhibiting inflammation. Herein, we tested whether EGCG participates in modulating cardiac ischemia reperfusion-induced injury and elucidate its potential mechanisms.

**Methods:**

To induce MI in mice, we employed coronary artery ligation, while cell models utilized oxygen glucose deprivation/re-oxygenation (OGD/R)-treated HL-1 cells. TTC, HE and Massion staining evaluated the pathological changes of heart tissues. Besides, RNA-pull down and RIP assays analyzed the interactions of MEG3/TAF15 and AIM2 mRNA/TAF15. FISH associated with immunofiuorescence (IF) double staining was conducted to measure the co-localization of MEG3 and TAF15.

**Results:**

In vitro and in vivo evidence supported that EGCG treatment improved cardiomyocytes viability while inhibiting the expressions of AIM2, C-caspase-1, ASC, GSDMD-N, IL-18 and IL-1β. Knockdown of MEG3 intensified EGCG's therapeutic effects both in vitro and in vivo. LncRNA MEG3 and AIM2 mRNA interacted with TAF15, and MEG3, in turn, promoted the stability of AIM2 mRNA through regulating TAF15. Overexpression of TAF15 reversed the promoting effect of EGCG and MEG3 knockdown on cell viability, and the inhibiting effect on cell pyroptosis.

**Conclusion:**

EGCG protected cardiomyocytes from pyroptosis by the MEG3/TAF15/AIM2 axis, indicating EGCG as a potential novel therapeutic strategy for managing MI.

**Supplementary Information:**

The online version contains supplementary material available at 10.1186/s13020-023-00856-z.

## Introduction

Myocardial infarction (MI) stands as a prevalent and lethal cardiovascular event [1]. MI leads to progressive damage to the heart muscle, culminating in heart failure. This affliction is conventionally linked to the protracted ischemic circumstances arising from reduced coronary blood flow and oxygen delivery to the heart. On the cellular level, MI engenders manifold deleterious consequences for cardiomyocytes, including cell apoptosis, necrosis, and inflammation [2]. Given that cardiomyocytes are terminally differentiated cells that cannot regenerate, preserving their viability and function assumes great significance in MI management.

( −)-Epigallocatechin-3-gallate (EGCG) is main bioactive polyphenol isolated from green tea that has been reported to possess the abilities of antioxidant, anti-atherosclerosis, anti-inflammation, and free radical cleaning [3]. Evidence has substantiated the potential of EGCG to confer a broad spectrum of advantages to cardiovascular health through diverse mechanism [4]. Both in vitro and in vivo investigations have illustrated that EGCG could reduce myocardial fibrosis by regulating endoglin [5]. Recent findings have underscored that EGCG protected cardiomyocytes against MI by regulating Wnt3a/β-catenin signaling pathway through miR-145/Dab2 axis [6]. Meanwhile, EGCG has demonstrated the capability to ameliorate anxiety-related behaviors in MI-afflicted rats and reduced IL-6 levels in serum and hippocampus [7]. However, the underlying mechanisms of EGCG in MI remain inadequately elucidated, necessitating further comprehensive inquiry.

Long non-coding RNAs (lncRNAs) are a subfamily of non-protein coding transcripts characterized by lengths over 200 nucleotides [8], and have been ascribed multifaceted roles in biological processes, encompassing inflammation, differentiation, and cell development [9]. Among them, lncRNA maternally expressed gene 3 (MEG3), an imprinted lncRNA with a human length of 1.6 kb, has emerged as a significant focus in MI research. Investigations have indicated that MEG3 knockdown showed protective role against cardiac fibrosis and diastolic performance through regulating matrix metalloproteinase-2 (MMP-2) in vitro and in vivo [10]. However, whether MEG3 plays a role in cardiomyocyte injury during MI remains unknown.

Pyroptosis, a form of programmed cell death accompanied by inflammation, has been linked with cell apoptosis [11]. Intriguingly, the expressions of key pyroptosis-related genes, such as GSDMD, and caspase-1, were upregulated in MI models [12], indicating a significant role for pyroptosis in the progression of MI. Pyroptosis is initiated by the activation of inflammasomes, including NACHT, absent in melanoma 2 (AIM2), or pyrin [13], which regulate caspase-1 activation and the release of several pro-inflammatory cytokines such as IL-1β and IL-18. In an MI mouse model induced by ligation of the left anterior descending coronary artery, AIM2, caspase-1, and IL-18 were upregulated, and cardiomyocyte death occurred in peri-infarct or infarct regions of the left ventricle [14], suggesting the high AIM2 inflammasome activities in MI. However, the specific mechanism needs further investigation, particularly regarding whether MEG3 affects pyroptosis regulated by AIM2 inflammasome, which has not yet been reported.

Previous studies have shown that lncRNAs can function as competing endogenous RNAs (ceRNAs) or directly interact with proteins directly to regulate mRNAs. TATA-box binding protein associated factor 15 (TAF15), an RNA binding protein (RBP), has been implicated in the progression of several tumors. For instance, Linc00504 recruited TAF15 to stabilize CPEB2 mRNA, thereby affecting the radio-sensitivity of breast cancer [15]. In this context, the data from StarBase (StarBase, https://starbase.sysu.edu.cn/index.php) predicted that MEG3 and AIM2 mRNA could bind with TAF15. We therefore hypothesized that MEG3 enhanced the stability of AIM2 mRNA through interacting with TAF15, thereby activating AIM2 inflammasome and promoting pyroptosis-mediated MI. Interestingly, EGCG treatment inhibited AIM2 activity by downregulating MEG3, leading to the alleviation of MI.

## Materials and methods

### Construction of MI mice model

After anesthetization, male C57BL mice were incubated and ventilated with a rodent ventilator. Then, thoracotomy and pericardiotomy were performed followed by left anterior descending (LAD) coronary artery ligation. The sham group mice underwent the same operations, except that LAD coronary artery ligation was not performed. After 4 weeks, induction of heart failure was confirmed by echocardiography, and mice were sacrificed. All studies were approved by the Committee for Animal experimentation and fulfilled the requirements for humane animal care from Tangdu Hospital, Air Force Medical University. For the experimental group, the model mice were treated intragastrically (i.g.) with EGCG (50 mg/kg) once daily by oral gavage under the condition transfected with or without lentivirus of shMEG3.

### Measurement of myocardial injury

A Vivid 7 Dimension echocardiography machine was performed on isoflurane-anesthetized mice to evaluate cardiac function. Left ventricular ejection fraction (LVEF), EF, and fractional shortening (FS) were measured as previously reported [16].

Creatine kinase-MB (CK-MB) and lactate dehydrogenase (LDH), the biomarkers of tissue damages including myopathy and MI, were detected by corresponding ELISA kits.

### Collection of tissues

Treated mice were sacrificed after being sedated with pentobarbital sodium (50 mg/kg) administered intraperitoneally. The mice chest was opened, the mice heart was removed, and the atria, large blood vessels and the hoof tissue of the heart were removed. And the tissue was obtained, and fixed.

### TTC staining

The hearts of mice in different groups were frozen at a −80 °C refrigerator for 8 min, then cut into five thick circular slices, respectively (0.1–0.3 cm). Afterwards, the slices were immersed in prepared TTC solution and cultured at 37 °C for half of hour (protected from light), followed by incubating in 10% formaldehyde for a week. Finally, slices were arranged from big to small in order and photographs were acquired by Sony camera to observe infarction size of myocardial.

### Hematoxylin and eosin (H&E) staining

After fixation in 4% paraformaldehyde, heart tissues from each group were washed and embedded in paraffin, followed by slicing with a microtome into 5 μm sections. After dewaxing and dehydrating, slices were stained with hematoxylin and eosin. Slices were then imaged using a microscope (Olympus, Tokyo, Japan).

### Masson’s trichrome staining

Formaldehyde-fixed paraffin-embedded tissues (5 µm sections) were used for Masson’s trichrome stain to detect the interstitial fibrosis. The collagen fibers stains blue, the cytoplasm stains red, and the nuclei stains black.

### Immunohistochemistry (IHC)

Deparaffinized and rehydrated sections were boiled in Na-citrate buffer for antigen retrieval, followed by incubating with anti-AIM2 (1:500, ab93015, Abcam) at a dilution of 1:200 overnight. Afterward, slices counterstained with hematoxylin, dehydrated and coverslipped. Images were acquired by a microscope at a magnification of 200.

### Cell culture and establishment of in vitro myocardial I/R model

HL-1 and 293 T cells offered by Cell Bank of Chinese Academy of Sciences (Shanghai, China) were kept in DMEM supplemented with 10% fetal bovine serum, 100 μg/mL streptomycin and 100 U/mL penicillin (all from Gibco, Carlsbad, USA) with 5% CO_2_ at 37 °C. Myocardial I/R model was constructed through oxygen–glucose deprivation/reperfusion (OGD/R) stimulation. Briefly, HL-1 cells were suspended in sugar-free DMEM and cultured in an anoxic chamber containing 95% N2 and 5% CO_2_ for 2, 4, 8, or 12 h, followed by maintenance in normal culture condition for 24 h as reperfusion. HL-1 cells were incubated with EGCG (0, 5, 10, 20, 40, 80 or 100 μM, Sigma Aldrich, St Louis, MO, USA; E171) for three hours prior to OGD/R treatment.

### Cell transfection

Short hairpin RNA (shRNA) targeted MEG3, TAF15, FUS, EIF4A3, DGCR8, EWSR1, ELAVL1 (shMEG3, shTAF15, shFUS, shEIF4A3, shDGCR8, shEWSR1, shELAVL1) or scrambled oligonucleotides purchased from GenePharma (Shanghai, China) were inserted into pGLVH1 vector. The MEG3 or TAF15 fragments were cloned into pcDNA3.1 (Invitrogen, Carlsbad, CA, USA) vector (oe-MEG3, oe-TAF15). Lipofectamine 3000 was adopted to conduct the transfection of the above vectors into HL-1 cells. All sequences related to transfection were shown as follow: shMEG: 5'- CACCGCAGAAACCAGTATTGAAATGCGAACATTTCAATACTGGTTTCTGC -3';

shTAF15: 5'- CACCGGACAAACACCACAAGGTTATCGAAATAACCTTGTGGTGTTTGTCC -3';

shFUS: 5'- CACCGCAGCTATGGTTCTTCTTATGCGAACATAAGAAGAACCATAGCTGC -3';

shEIF4A3: 5'-CACCGCAGCAGCGTGCTATCAAGCAGATAACGAATTATCTGCTTGATAGCACGCTGCTG-3';

shDGCR8: 5'-CACCGGAGACATATGAGAGTCCCTCTCCTCGAAAGGAGAGGGACTCTCATATGTCTCC-3';

shEWSR1: 5'-CACCACAGTGCAATTTATGTGCAAGGATTCGAAAATCCTTGCACATAAATTGCACTG-3';

shELAVL1: 5'-CACCGGATGACATTGGGAGAACGAATTTACGAATAAATTCGTTCTCCCAATGTCATCC-3';

For silencing MEG3 in mouse model, the adeno-associated virus serotype 9 (AAV9) vectors carrying a shMEG3 were constructed as described previously [17]. Mouse model received the virus solution (2 × 10^11^ genome-containing particles (GC)/animal) via tail vein injection.

### Nuclear-cytosol fractionation

The NE-PER Nuclear Cytoplasmic Extraction Reagent kit (Thermo Fisher Scientific, Waltham, MA, USA) was used for nucleocytoplasmic separation. Briefly, suspended cells were centrifuged at, followed by discarding the supernatant. Subsequently, cytoplasmic extraction reagent I, cytoplasmic extraction reagent II, and nuclear extraction reagent were added sequentially according to the protocols to obtain nuclear and cytoplasmic extracts. Finally, trizol was added to extract nuclear and cytoplasmic RNAs, and qRT-PCR was used to further analyze the expression of MEG3, GAPDH, and U6 in the nucleus and cytoplasm.

### Measurement of mRNA stability

Traeted cells were seeded in 6-well plates and treated with actinomycin-D (act-D), which was added to inhibit further RNA synthesis at a final concentration of 5 μg/mL, for 0, 1, 2, 4, or 8 h. Total RNA was extracted by Trizol reagent and analyzed by qRT-PCR.

### CCK-8

Treated cells were plated into 96-well plates (5000 cells per well) and cultured for 48 h. Next, CCK-8 reagent (10 µL) was added to each well and incubated with cells for 1 h at 37 °C. At 48 h post-transfection, cell viability was detected at 450 nm by a spectrophotometer (BioRad, Hercules, CA, USA). The results represent the mean of three replicates under the same conditions.

### TUNEL assay

DNA fragmentation in HL-1 cells were measured by the DeadEnd™ Fluorometric TUNEL System (Promega, Madison, WI, USA; G3250). Cells were stained by DAPI to identify the nuclei followed by visualization under fluorescence microscope. TUNEL-detected DNA stains green, and overlapped green and blue nuclear fluorescence indicates DNA fragmentation due to cell death.

### Quantitative real-time PCR (qRT-PCR)

Total RNAs of treated tissues or cells were extracted with Trizol reagent (Invitrogen), and then was reverse-transcribed to cDNA with Prime-Script RT-PCR master mix (Takara, Tokyo, Japan). RT-qPCR detection was performed with SYBR Green qPCR (Applied Biosystems, Carlsbad, CA, USA; 4309155). β-actin was served as internal controls for mRNA. The gene expression levels were presented as fold changes relative to the expression levels of appropriate controls using the 2^–ΔΔCt^ method. The following primer sets were used: MEG3, forward (5ʹ–3ʹ) TCCTCACCTCCAATTTCCCCT, reverse (5ʹ–3ʹ) GAGCGAGAGCCGTTCGATG; AIM2, forward (5ʹ–3ʹ), AAAACTGCTCTGCTGCCCT, reverse (5ʹ–3ʹ); TCAGCACCGTGACAACAAGT; TAF15, forward (5ʹ–3ʹ), GGGGAAGCAACAGTGTCATT, reverse (5ʹ–3ʹ); AAAACCTCCACGGCCTCTAT; β-actin, forward (5ʹ–3ʹ) GGCTGTATTCCCCTCCATCG, reverse (5ʹ–3ʹ) CCAGTTGGTAACAATGCCATGT;

### Western blot analysis

After isolating total proteins using RIPA buffer (Invitrogen), fifty-gram proteins of each group were subjected for purification using 10% SDS–polyacrylamide gels. Afterwards, transferred the purified proteins to PVDF membranes followed by blocking non-specific sites using 5% skim milk. The membranes were then subjected to primary antibodies incubation overnight at 4 °C, and corresponding HRP- conjugated secondary antibody (1:5000, sc-2004, Santa Cruz Biotechnology) were used to probe the related primary antibodies. The antibody-reactive bands were detected with ECL reagent (Millipore Corp, Billerica, MA, USA). The following primary antibodies were used: anti-AIM2 (1:1000, ab93015; Abcam, Cambridge, MA, UK), anti-ASC (1:2000, ab180799, Abcam), anti-C-caspase-1 (1:1000, ab179515, Abcam), anti-GSDMD-N (1:2000, PA5-116815, Thermo Fisher), anti-IL-18 (1:2000, ab52914, Abcam), anti-IL-1β (1:1000, ab254360, Abcam), and anti-TAF15 (1:10000, ab134916).

### Immunofluorescence staining

After 15 min of fixation in 10% formalin, HL-1 cells were incubated with PBS containing 5% BSA for 1 h, followed by overnight incubation with with primary antibody against AIM2 (1:100, ab93015, Santa Cruz Biotechnology) at 4 °C. For immunofluorescence staining, the cells were then incubated with secondary antibodies with Alexa Fluor^®^ 488 (anti-mouse IgG, 1:100, sc-516248, Santa Cruz Biotechnology) for 1 h at RT. DAPI was used as a control for staining the nucleus. The FV-1200 laser scanning confocal microscope was used for visualization and the results were analyzed by Image J.

### RNA-pull down

RNA pull-down was performed to analyze the interplay between MEG3 and TAF15, or AIM2 mRNA and TAF15. Biotin-labeled MEG3 and AIM2 were designed and provided by GenePharm (Shanghai, China). Biotin-labeled MEG3 and AIM2 or their anti-sense RNAs were transcribed and purified, followed by mixing and incubating with the cell lysates overnight. Then, streptavidin-conjugated magnetic beads (Invitrogen) were added and allowed for another 2 h incubation. Beads were then washed thoroughly, and the retrieved proteins were examined using western blot.

### RNA immunoprecipitation (RIP)

RIP assay was conducted to analyze the interplay between MEG3/TAF15 and AIM2 mRNA/TAF15 using the Magna RIP kit (EMD Millipore, USA). Cells were harvested and lysed in the complete RIP lysis buffer. Then, lysate was incubated with magnetic beads conjugated with anti-TAF15 or IgG (used as control). Co-precipitated RNAs were extracted, enriched and detected using RT-qPCR. Total RNAs (input control) were also detected.

### FISH and immunofluorescence (IF) double staining

The MEG3 and TAF15 FISH probes were designed and supplied by Genema (Shanghai, China). Cells were seeded on coverslips and allowed to growth for 24 h, followed by the fixation with 4% paraformaldehyde. Next, cells were incubated with the MEG3 or TAF15 probe (1: 50) overnight in PBS containing 0.5% Triton X-100, blocked with 0.07% streptavidin solution. Afterwards, the cells were incubated with anti-TAF15 (1:500, ab134916, Abcam) overnight, followed by the incubation with Alexa Fluor 594 conjugated secondary antibody for 2 h and stained with 4′, 6-diamidino-2-phenylindole (DAPI). Signals were observed under a confocal microscope (Olympus, Japan).

### Statistical analysis

All data were shown as mean ± SEM. Statistical analysis of the results was performed by a student’s *t*-test (two groups) or one-way ANOVA (multiple groups). P < 0.05 was regarded as statistically significant.

## Results

### EGCG alleviated MI in the in vivo mice model

**Fig. 1:**
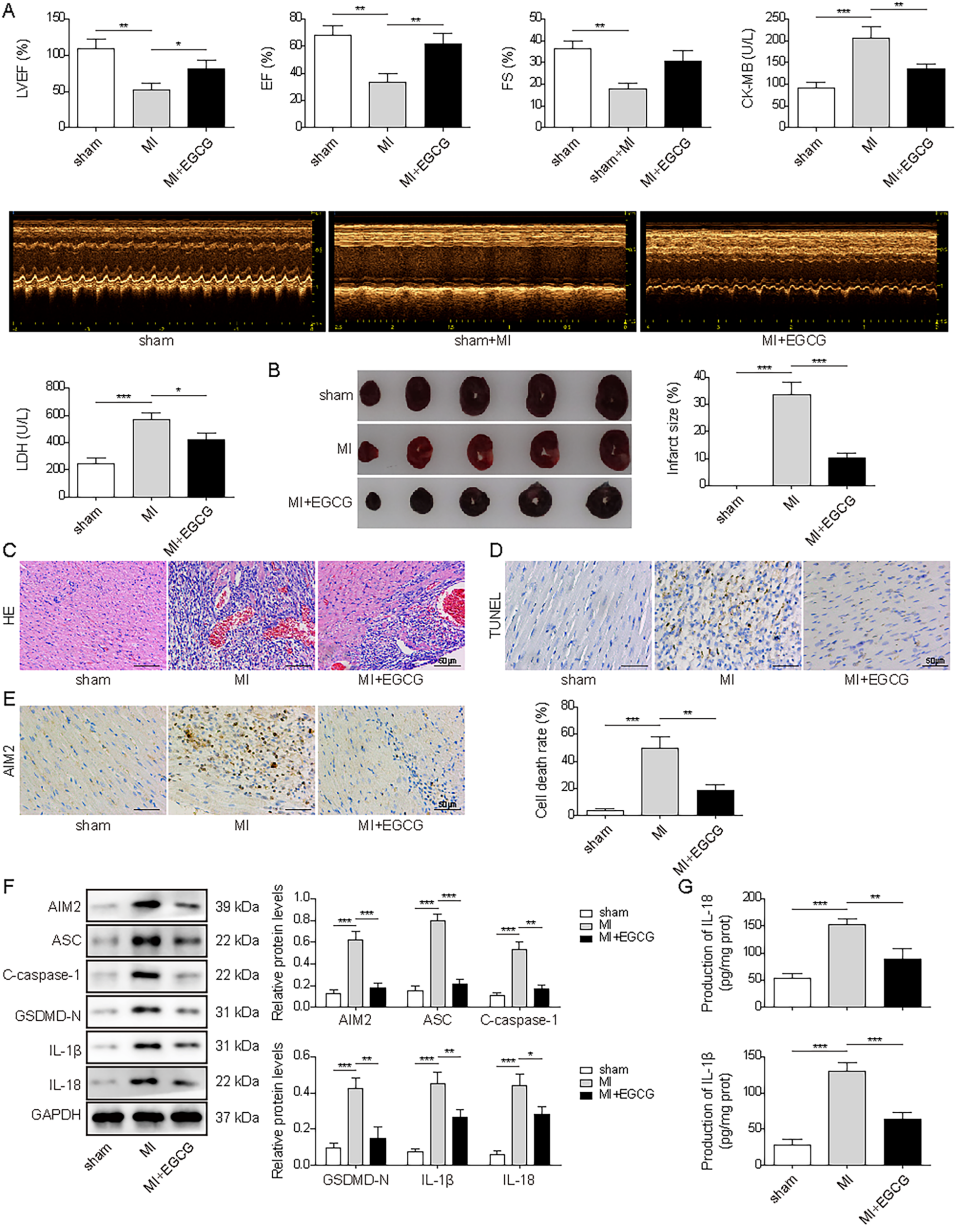
EGCG alleviated MI in the in vivo model. MI model mice were treated with EGCG (50 mg/kg) and divided into three groups: sham, MI and MI + EGCG. N = 8. **A**. Cardiac function was assessed by echocardiography analysis and measurement of CK-MB and LDH. **B**. TTC staining evaluated the infarction size of myocardial of mice in each group. **C**. The degree of myocardial changes of heart tissues was analyzed by HE staining (scale bar = 50 µm). **D**. TUNEL detected the cell death (scale bar = 50 µm). **E**. IHC measured the expression of AIM2 (scale bar = 50 µm). **F**. Western blot analysis of AIM2 (39 kDa), C-caspase-1 (22 kDa), ASC (22 kDa), GSDMD-N (31 kDa), IL-18 (22 kDa), and IL-1β (31 kDa). β-actin (42 kDa) was used as a loading control. **G**. Relative production of IL-18 and IL-1β. *p < 0.05, **p < 0.01, ***p < 0.001

To investigate the function of EGCG in vivo, MI mouse models were constructed and administrated with EGCG. Echocardiography analysis indicated that compared to the sham group, MI mice exhibited reduced LVEF, EF, and FS, however, EGCG treatment increased these levels in MI mice (Fig. 1A). Additionally, MI mice showed elevated levels of CK-MB and LDH. Treatment with EGCG restored CK-MB and LDH levels to some extent (Fig. 1A). Moreover, EGCG treatment inhibited the increased infarction size in the myocardium of MI mice (Fig. 1B) and mitigated the extent of myocardial changes (Fig. 1C). TUNEL assay results indicated increased cell death in MI mouse models, but this effect was blocked by EGCG treatment (Fig. 1D). Pyroptosis may have been triggered by the activation of AIM2 inflammasome, which regulates caspase-1 activation and the release of several pro-inflammatory cytokines such as IL-1β and IL-18 [13]. To confirm the impacts of EGCG on pyroptosis, we then tested the expression of AIM2 and pyroptosis-related proteins. The results indicated that AIM2 was upregulated in MI mice, but EGCG treatment reduced AIM2 expression (Fig. 1E). As expected, EGCG downregulated the elevated expression of C-caspase-1, ASC, GSDMD-N, IL-18, and IL-1β in MI mice (Fig. 1F and G). These findings suggested that EGCG could partially alleviate the progression of MI in vivo by inhibiting pyroptosis.

### EGCG attenuated the cardiomyocyte pyroptosis induced by OGD/R

**Fig. 2:**
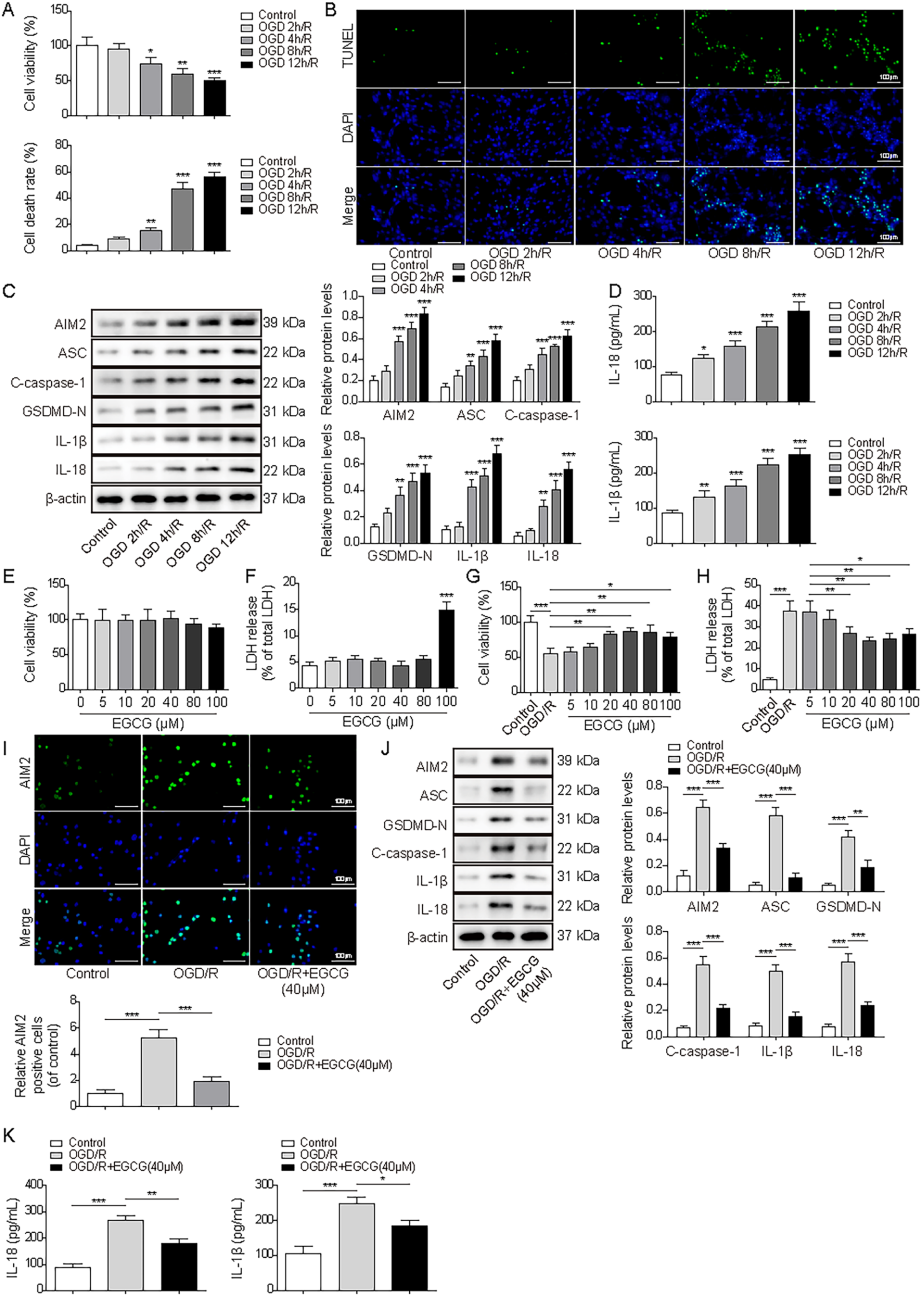
EGCG attenuated the cardiomyocyte pyroptosis induced by OGD/R. HL-1 cells were exposed to OGD/R for indicated time (OGD for 0, 2, 4, 8 or 12 h) and divided into five groups: Control, OGD 2 h/R, OGD 4 h/R, OGD 8 h/R, and OGD 12 h/R. **A** and **B**. CCK-8 and TUNEL assay assessed the viability and death rate of cells in different groups. (scale bar = 100 µm). **C**. Protein expressions of AIM2, C-caspase-1, ASC, GSDMD-N, IL-18 and IL-1β. **D**. Relative production of IL-18 and IL-1β in each group. HL-1 cells were treated with EGCG (0, 5, 10, 20, 40, 80, 100 μM), and then **E** cell viability and **F** release of LDH were measured. OGD/R cells were treated with different concentration of EGCG, then the **G** the cell viability and **H** the release of LDH were detected. Cells were exposed to OGD/R before treating with EGCG (40 μM), and then the expression of AIM2 were evaluated by **I** Immunofluorescence (scale bar = 100 µm). **J** Protein expression of AIM2, C-caspase-1, ASC, GSDMD-N, IL-18 and IL-1β in each group. **K** IL-18 and IL-1β relative levels were tested by corresponding ELISA kits. The results were represented by three individual experiments. *p < 0.05, **p < 0.01, ***p < 0.001

To investigate the potential mechanism of EGCG in improving MI in vitro, we subjected HL-1 cells to varying durations of OGD (2, 4, 8, or 12 h) followed by reoxygenation (24 h) before administering EGCG treatment. With the extension of OGD treatment time, we observed a decreased in cell ability and an increased in cell death (Fig. 2A and B). Furthermore, in order to corroborate the influence of OGD/R exposure duration on cellular pyroptosis, the expressions of AIM2, C-caspase-1, ASC, GSDMD-N, IL-18, and IL-1β were evaluated. Our findings revealed that the expressions of these markers increased proportionally with the extension of the OGD treatment period (Fig. 2C and D). Based on these observations, we determined that an 8-h OGD followed by 24-h re-oxygenation regimen induced a close to 50% reduction in cell viability and pronounced alterations in cell pyroptosis. Consequently, this protocol was adopted to establish cell models for subsequent experiments. Prior to subsequent experiments, we conducted an assessment of the potential cytotoxicity of EGCG on HL-1 cells. Cells were treated with different concentration of EGCG (0, 5, 10, 20, 40, 80 or 100 μM). The results suggested that EGCG treatment did not exert a significant impact on cell viability (Fig. 2E). The evaluation of LDH levels is vital in assessing cellular injury. As shown in Fig. 2F, LDH levels remained relatively stable (except for the group treated with 100 μM EGCG). These findings collectively suggested that EGCG was not toxic for HL-1 cells. Subsequently, we observed that EGCG treatment could promote the viability of OGD/R cells and suppress the LDH levels. Notably, EGCG at a concentration of 40 μM exhibited the most favorable effects on OGD/R cells, yielding the highest cell viability and the lowest LDH levels (Fig. 2G and H). Furthermore, EGCG treatment decreased the upregulated expressions of AIM2, C-caspase-1, ASC, GSDMD-N, IL-18 and IL-1β in OGD/R cells (Fig. 2I–K). Therefore, EGCG treatment inhibited cardiomyocyte pyroptosis induced by OGD/R, potentially by downregulating AIM2 levels.

### EGCG attenuated cardiomyocyte pyroptosis mediated by AIM2 through downregulating MEG3 in OGD/R cells

**Fig. 3:**
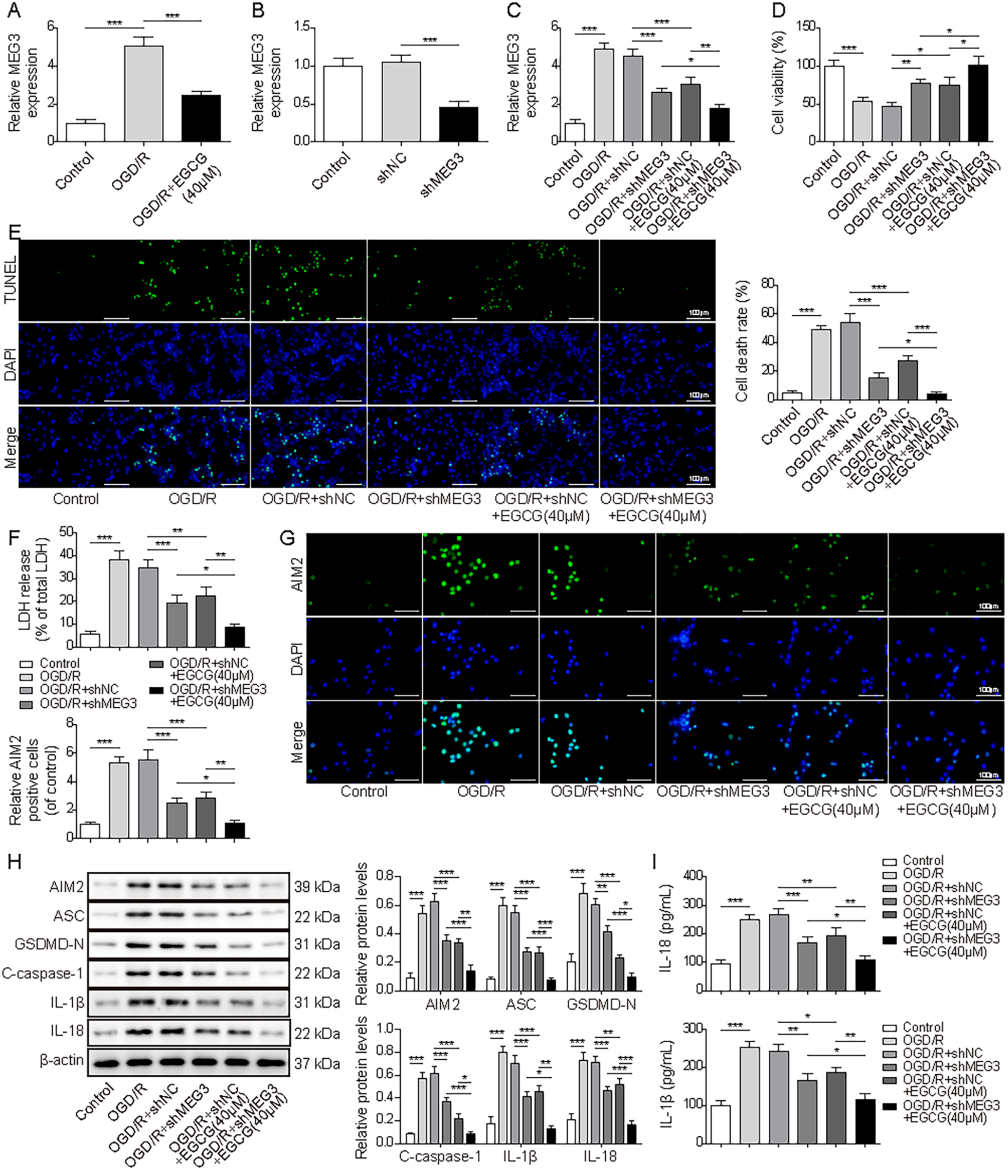
EGCG attenuated cell pyroptosis mediated by AIM2 through down-regulating MEG3 in OGD/R cells. **A**. Relative expression of MEG3 in OGD/R cells with and without EGCG treatment. **B**. Validation of the knockdown efficiency of shMEG3 in HL-1 cells. Cells were exposed to OGD/R before transfecting with shMEG3 or treating with EGCG (40 μM) and divided into six groups: control, OGD/R, OGD/R + shNC, OGD/R + shMEG3, OGD/R + shNC + EGCG, OGD/R + shMEG3 + EGCG. **C**. The measurement of MEG3 levels. **D** and **E** The analysis of cell viability and death (scale bar = 100 µm). **F** The measurement of LDH levels. **G**. Immunofluorescence assay was used to evaluate AIM2 levels. (scale bar = 100 µm). **H** Protein levels of AIM2, C-caspase-1, ASC, GSDMD-N, IL-18 and IL-1β. **I**. The expressions of IL-18 and IL-1β was detected by ELISA. The results were represented by three individual experiments. *p < 0.05, **p < 0.01, ***p < 0.001

MEG3 have been implicated to be associated with myocardial injuries. Herein, we explored the potential impact of EGCG on MEG3 regulation. EGCG downregulated the elevated levels of MEG3 in OGD/R cells (Fig. 3A). Following shMEG3 transfection, MEG3 levels decreased by approximately 50%, indicating that MEG3 was successfully knocked down in HL-1 cells (Fig. 3B). Subsequent results revealed that the increased MEG3 induced by OGD/R was inhibited by silencing MEG3, and EGCG treatment further reduced its levels (Fig. 3C). Furthermore, OGD/R induced reduced cell viability and increased cell mortality were counteracted by MEG3 knockdown or EGCG treatment, both interventions increased cell viability while repressing cell death, demonstrating a synergistic effect (Fig. 3D and E). The increased release of LDH in OGD/R cells was also mitigated by MEG3 knockdown or EGCG treatment (Fig. 3F). The upregulated AIM2, C-caspase-1, ASC, GSDMD-N, IL-18 and IL-1β in OGD/R cells were inhibited after knockdown of MEG3 or treatment with EGCG, and their suppression was even more pronounced when MEG3 knockdown and EGCG treatment were combined (Fig. 3G–I). In conclusion, EGCG treatment regulated AIM2 inflammasome pathway through downregulating MEG3 levels, thereby inhibiting cardiomyocyte pyroptosis.

### The interaction between MEG3 and TAF15.

**Fig. 4:**
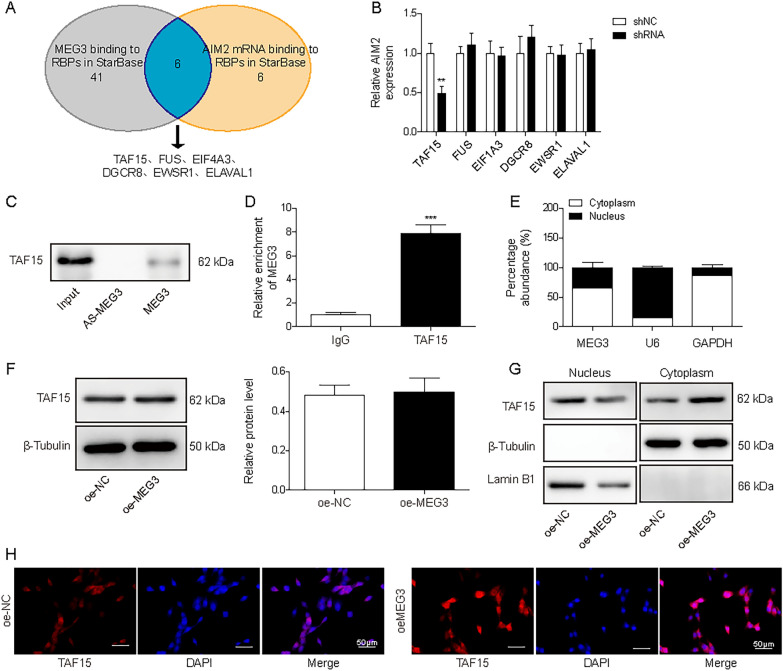
The interaction between MEG3 and TAF15. **A**. Six potential targeted RNA-binding proteins (RBPs) of MEG3 (TAF15 (62 kDa), FUS (53 kDa), EIF4A3 (47 kDa), DGCR8 (86 kDa), EWSR1 (68 kDa), and ELVAL1 (36 kDa)) were predicted by StarBase. **B**. The verification of RBPs through evaluating AIM2 levels in HL-1 cells silenced potential targeted RBPs. **C** and **D**. RNA pull-down and RIP were conducted to prove the interplay between MEG3 and TAF15. **E**. The distribution of MEG3 in cytoplasm and nucleus. **F**. Protein expression of TAF15 in HL-1 cells with MEG3 overexpression. **G**. The expression of TAF15 in nucleus and cytoplasm. **H**. FISH and IF double staining indicated the co-localization of MEG3 and TAF15. (scale bar = 50 µm). The results represent three individual experiments. **p < 0.01, ***p < 0.001

We initiated our subsequent investigation by identifying potential RBPs of MEG3. Figure 4A illustrates the use of StarBase for RBP prediction, which identified six potential RBPs: TAF15, FUS, EIF4A3, DGCR8, EWSR1 and ELVAL1. The knockdown plasmids were used to silence the expression of these proteins and the knockdown efficiencies were depicted in Additional file 1: Figure S1A–F. Interestingly, we observed a significant suppression of AIM2 expression after transfecting with shTAF15 (Fig. 4B). Consequently, TAF15 was selected for subsequent experiments. The data from RNA-pull down indicated that biotin-labeled MEG3 precipitated TAF15 in cell lysates (Fig. 4C). A specific enrichment of MEG3 and TAF15 was found through RIP assay (Fig. 4D). Subsequently, we observed the presence of MEG3 in both the cytoplasm and nucleus, with a predominant presence in the cytoplasm (Fig. 4E). It's worth noting that overexpression of MEG3 did not change the expression of TAF15 (Fig. 4F). Then we measured the distribution of TAF15 in cytoplasm and nucleus and found that overexpression of MEG3 promoted the translocation of TAF15 from nucleus to cytoplasm (Fig. 4G). The results from Fig. 4H indicated an increased co-localization of MEG3 and TAF15 in cytoplasm. These findings suggested that the combination of MEG3 and TAF15 could facilitate the translocation of TAF15 from nucleus to cytoplasm.

### LncRNA MEG3 regulated the stability of AIM2 mRNA through binding to TAF15

**Fig. 5:**
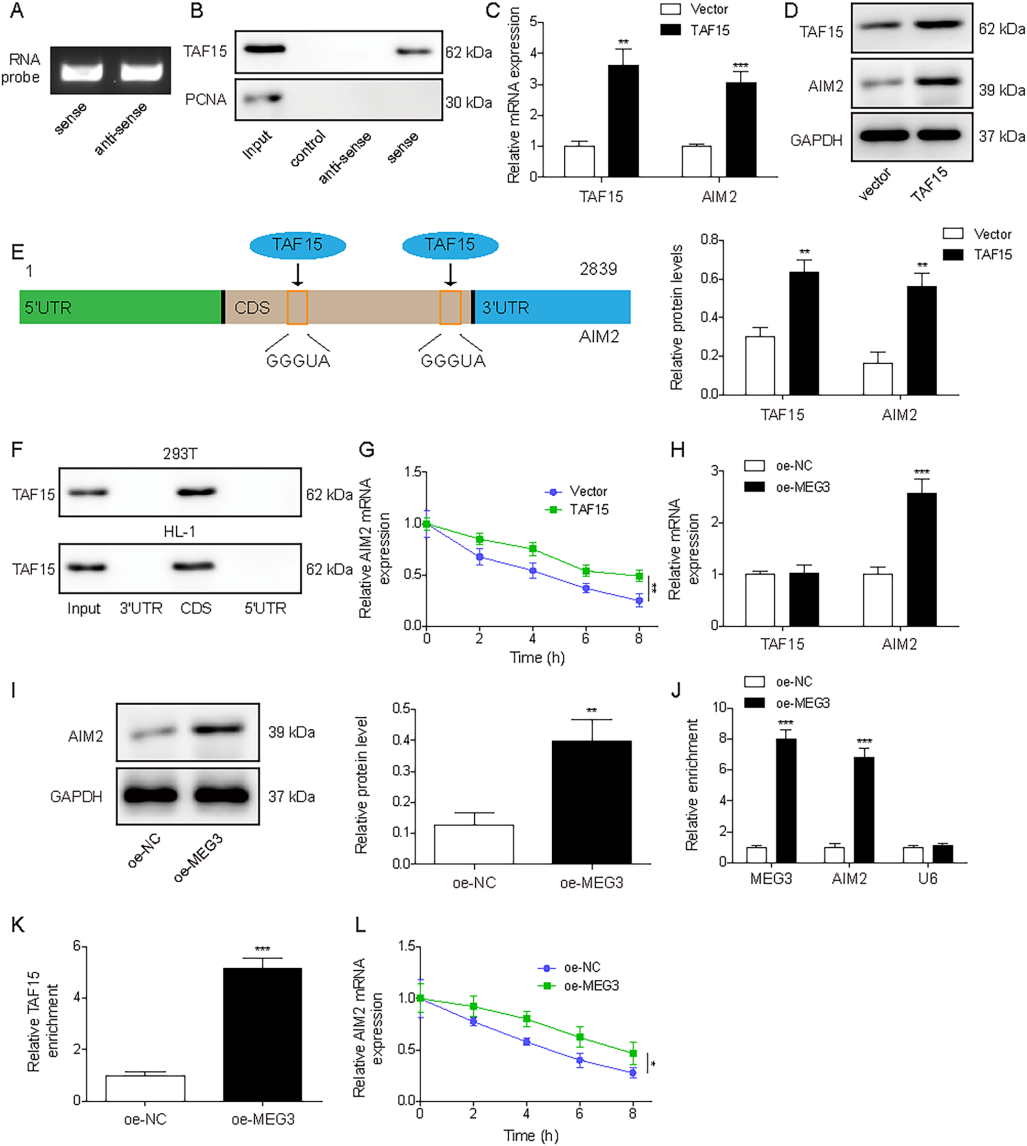
LncRNA MEG3 regulated the stability of AIM2 mRNA through binding to TAF15. **A**. A sense and an anti-sense RNA probe were prepared for AIM2 mRNA. **B**. RNA pull-down evaluated the interaction between TAF15 and AIM2 mRNA. **C** and **D**. Relative mRNA and protein expression of TAF15 and AIM2 in HL-1 cells overexpressed TAF15. **E**. StarBase analysis revealed TAF15-binding sequences in 5’-UTR, CDS, and 3’-UTR of AIM2 mRNA. **F**. Biotin-labeled probes corresponding to the CDS, 5’-UTR and 3’-UTR of AIM2 mRNA were generated. The corresponding interaction with TAF15 was examined by RNA pull-down assay in 293 T and HL-1 cells. **G**. The analysis of the stability of AIM2 mRNA through actinomycin D treatment (0, 2, 4, 6 or 8 h) in HL-1 cells overexpressed TAF15. **H**. RT-qPCR analysis of the expressions of TAF15 and AIM2 mRNA after overexpression of MEG3. **I**. Western blot analysis of the expressions of AIM2 after overexpression of MEG3. **J**. Enrichment of MEG3 and AIM2 mRNA in the TAF15 antibody or normal IgG-immunoprecipitated fractions after overexpression of MEG3. **K**. The interaction between TAF15 and AIM2 mRNA was analyzed by RIP assay after overexpression of MEG3. **L**. The stability of AIM2 mRNA in HL-1 cells transfected with MEG3 overexpression plasmids after actinomycin D treatment. The results were represented for three individual experiments. *p < 0.05, **p < 0.01, ***p < 0.001

Upon uncovering the regulatory impact of MEG3 on AIM2 and the interaction between TAF15 and AIM2 mRNA, our next objective was to investigate whether the stability of AIM2 was controlled by MEG3/TAF15 axis. Firstly, a sense and an anti-sense RNA probe were prepared for AIM2 mRNA (Fig. 5A), and TAF15 was found to bind with the sense of AIM2 mRNA (Fig. 5B). Subsequently, we observed a significant increase, more than threefold, in the mRNA level of TAF15 and a more than one fold increase in its protein levels after transfection with TAF15 (Fig. 5C and D). Overexpression of TAF15 also increased the mRNA and protein levels of AIM2 (Fig. 5C and D). StarBase analysis revealed TAF15-binding sequence (GGGUA) in 5’untranslated region (5’-UTR), coding sequences (CDS), and 3’-UTR of AIM2 mRNA (Fig. 5E). This was further confirmed through RNA-pull down assays, which revealed that TAF15 predominantly binds to the CDS of AIM2 mRNA (Fig. 5F). Given the interaction between TAF15 and AIM2 mRNA, we speculated that TAF15 might regulate AIM2 expression by affecting AIM2 mRNA stability. Indeed, we observed that TAF15 overexpression increased AIM2 mRNA levels after treating with act-D (Fig. 5G). As indicated in Additional file 1: Figure S1G, MEG3 levels increased significantly after transfecting with MEG3 plasmid. Intriguingly, overexpression of MEG3 increased AIM2 levels, but did not change the expression of TAF15 (Fig. 5H and I). Furthermore, overexpression of MEG3 enhanced the binding interaction between TAF15 and AIM2 mRNA (Fig. 5J and K) and increased the stability of AIM2 mRNA (Fig. 5L). Taken together, MEG3 contributed to the translocation of TAF15 from nucleus to cytoplasm, led to the interaction between TAF15 and AIM2 mRNA and facilitate AIM2 mRNA stability, thus promoting cardiomyocyte pyroptosis in vitro.

### EGCG inhibited AIM2-mediated cardiomyocyte pyroptosis through MEG3/TAF15 axis

**Fig. 6:**
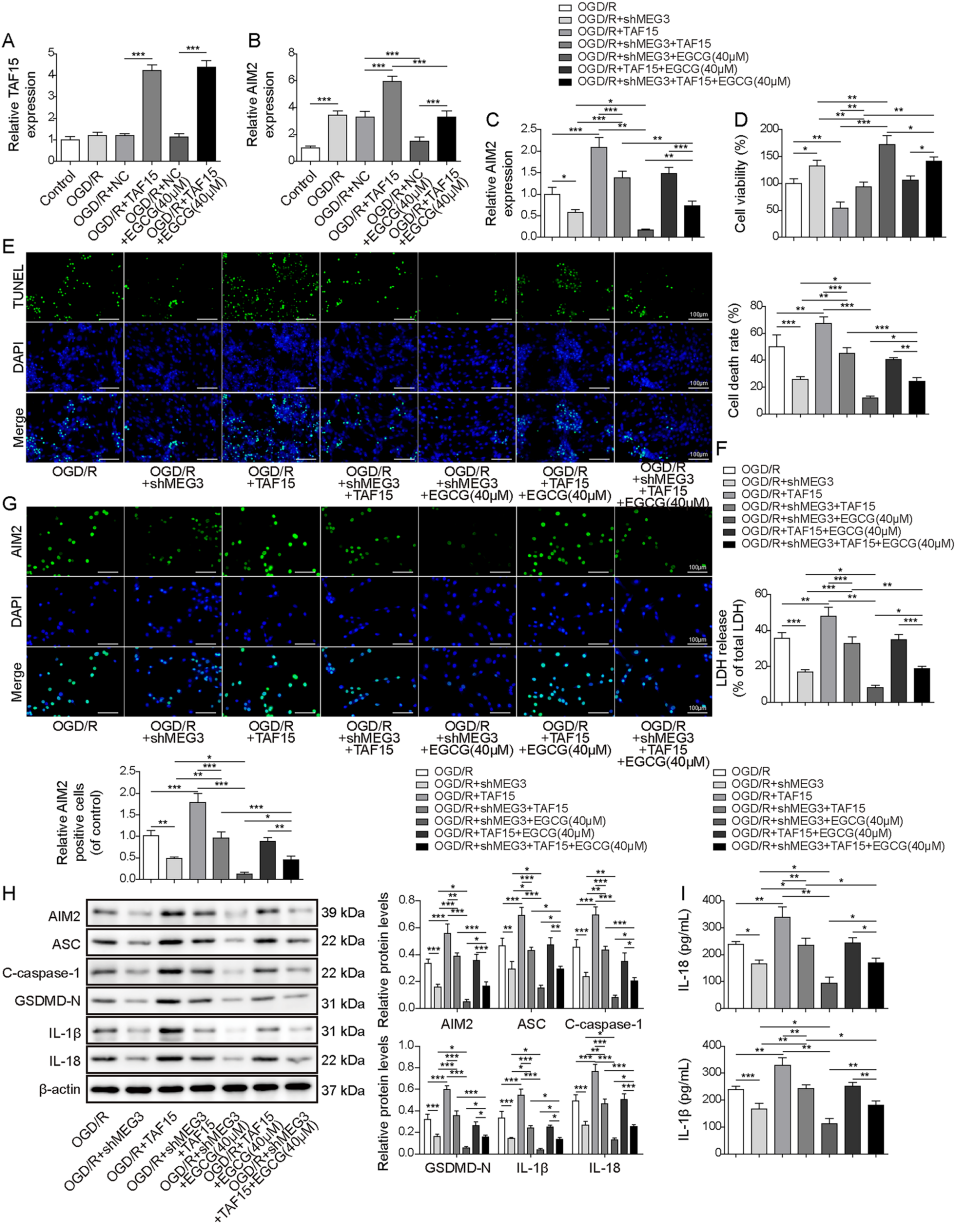
EGCG inhibited AIM2-mediated cardiomyocyte pyroptosis through MEG3/TAF15 axis. Cells were exposed to OGD/R before transfecting with TAF15 overexpression plasmids or treating with EGCG (40 μM) and divided into six groups: control, OGD/R, OGD/R + NC, OGD/R + TAF15, OGD/R + NC + EGCG and OGD/R + TAF15 + EGCG. **A** and **B**. The expressions of TAF15 and AIM2 were evaluated by RT-qPCR. Cells were exposed to OGD/R before transfecting with TAF15 overexpression plasmids or shMEG3 or treating with EGCG (40 μM) and divided into seven groups: OGD/R, OGD/R + shMEG3, OGD/R + TAF15, OGD/R + shMEG3 + TAF15, OGD/R + shMEG3 + EGCG, OGD/R + TAF15 + EGCG and OGD/R + shMEG3 + TAF15 + EGCG. **C**. The measurement of AIM2 levels. **D** and **E**. The cell viability and death were analyzed (scale = 100 µm). **F**. The levels of LDH were evaluated. **G**. Immunofluorescence assay was used to evaluate AIM2 levels. (scale bar = 100 µm). **H**. The expressions of AIM2, C-caspase-1, ASC, GSDMD-N, IL-18 and IL-1β were evaluated by Western blot. β-actin was used as a loading control. **I**. The expressions of IL-18 and IL-1β was detected by ELISA. The results were represented for three individual experiments. *p < 0.05, **p < 0.01, ***p < 0.001

To elucidate the biological function of MEG3/TAF15/AIM2 axis during the process of EGCG regulation of cardiomyocyte pyroptosis, rescue assays were designed and performed in this work. We observed that OGD/R treatment did not induce any significant alteration in the levels of TAF15. However, overexpression of TAF15 led to increased expression levels of both TAF15 and AIM2. Remarkably, EGCG treatment abrogated the upregulation of AIM2 induced by OGD/R or TAF15 overexpression (Fig. 6A and B). Next, knockdown of MEG3 attenuated the elevated AIM2 levels triggered by OGD/R, with EGCG treatment displaying a similar suppressive effect as MEG3 knockdown. Conversely, overexpression of TAF15 could reverse this inhibitory effect (Fig. 6C). Moreover, overexpression of TAF15 relieved the promoting effect of MEG3 knockdown or EGCG treatment on cell viability (Fig. 6D), and the inhibiting effect on cell death (Fig. 6E) and LDH levels (Fig. 6F). As expected, TAF15 overexpression upregulated the increased expressions of pyroptosis-related proteins induced by OGD/R, which were partially reversed by MEG3 knockdown or/and EGCG treatment (Fig. 6G–I). We therefore concluded that EGCG protected cardiomyocytes from pyroptosis by repressing MEG3/TAF15/AIM2 axis in vitro.

### EGCG repressed AIM2-mediated pyroptosis by downregulating MEG3 in vivo

**Fig. 7:**
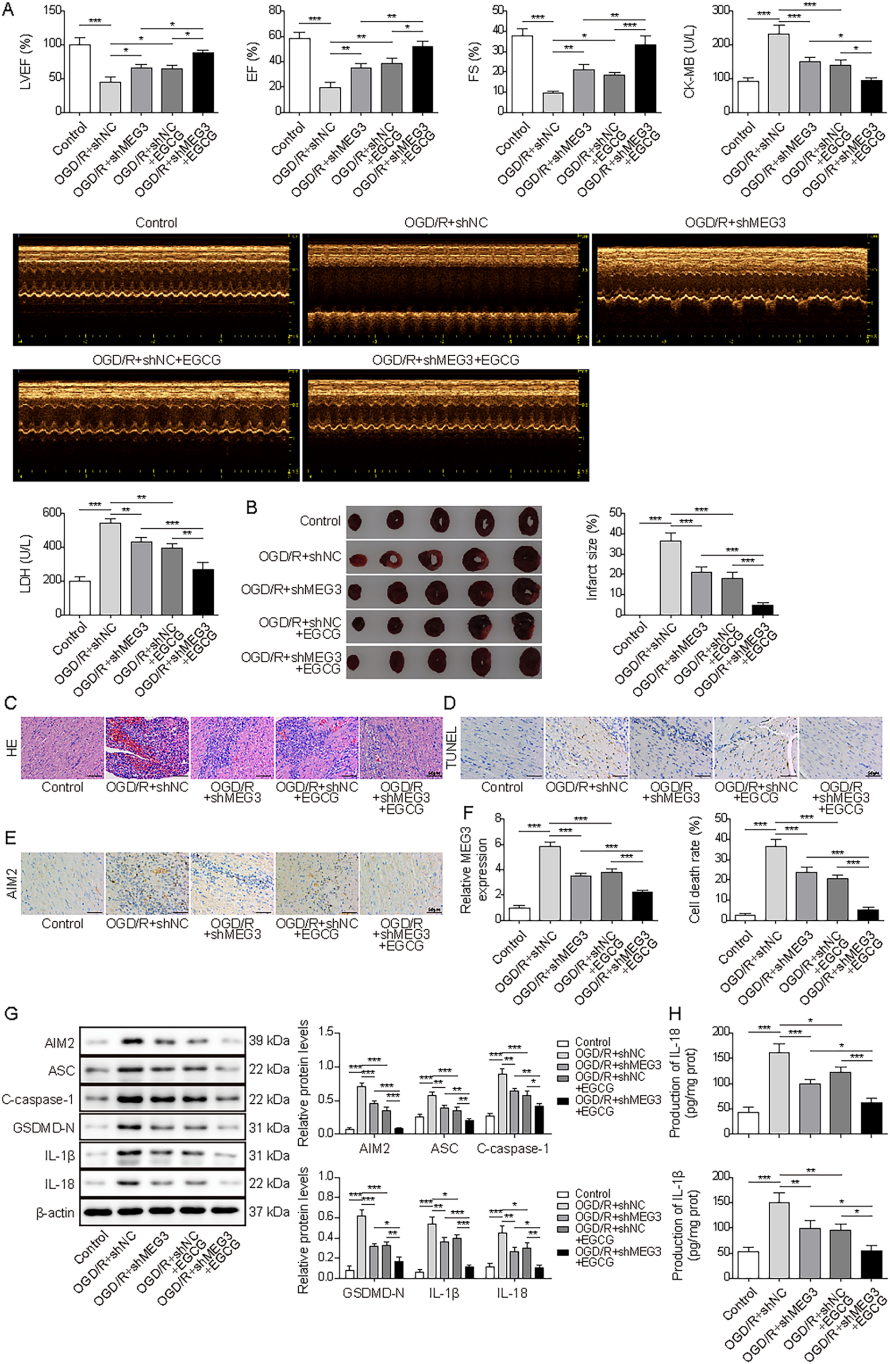
EGCG repressed AIM2-mediated pyroptosis by downregulating MEG3 in vivo*.* MI mice model was injected with lentivirus of shMEG3 to establish MEG3-silenced MI mice model, and treated with EGCG (50 mg/kg) and divided into five groups: sham, MI + shNC, MI + shMEG3, MI + shNC + EGCG and MI + shMEG + EGCG. N = 8. **A**. Cardiac function was assessed by echocardiography analysis and measurement of CK-MB and LDH. **B** and **C**. TTC and HE staining were employed to test myocardial infarction size and the degree of myocardial changes. (scale bar = 50 µm). **D**. TUNEL was used to analyze the cell death of heart tissues. (scale bar = 50 µm). **E**. IHC measured the expression of AIM2. (scale bar = 50 µm). **F**. RT-qPCR was performed to test MEG3 expression. **G**. Western blot analysis of expressions of AIM2, C-caspase-1, ASC, GSDMD-N, IL-18 and IL-1β. **H**. ELISA kits were used to analyze the production of IL-18 and IL-1β. *p < 0.05, **p < 0.01, ***p < 0.001

In vivo assays were subsequently conducted to further support our conclusions. MI mice were injected with AAV9-shMEG3 and then treated with EGCG. Subsequent analysis revealed a reduction in LVEF, EF, and fractional shortening FS, accompanied by elevated levels of CK-MB and LDH in the cardiac tissues of MI-afflicted mice. However, knockdown of MEG3 led to a discernible augmentation in both LVEF, EF, and FS percentages and a concurrent attenuation of CK-MB and LDH levels. Of paramount significance, the therapeutic application of EGCG further potentiated these salutary effects (Fig. 7A). Besides, knockdown of MEG3 or EGCG treatment demonstrated a pronounced capacity to impede myocardial infarction size, mitigate myocardial structural alterations, and mitigate cellular apoptosis within the MI models (Fig. 7B–D). The high expressions of AIM2 and MEG3 in heart tissues of MI mice were inhibited by silencing MEG3, and the effect was notably intensified following EGCG treatment (Fig. 7E and F). AIM2, C-caspase-1, ASC, GSDMD-N, IL-18 and IL-1β were overexpressed in MI mice, while MEG3 knockdown or EGCG treatment abolished these abnormal expressions (Fig. 7G and H). These findings indicated that EGCG inhibited AIM2-mediated cardiomyocyte pyroptosis through repressing MEG levels in vivo.

## Discussion

Current therapies for heart failure after MI are limited and non-curative. The inhibition of cardiomyocyte death help preserves cardiac tissue, reduce the extent of myocardial damage, and improve long-term cardiac function, ultimately contributing to better patient outcomes and reduced risk of heart failure post-MI [18]. As a polyphenol, EGCG is recognized for its diverse range of bioactivities [19]. It has been implicated in protection against ischemic myocardial injuries [20, 21]. However, the specific mechanisms of action for EGCG in MI remain elusive. The objective of this study is to elucidate the potential signaling pathway through which EGCG exerts its mitigating effects on myocardial injuries. To achieve this, EGCG was employed to treat with MI mouse and cell models and the results suggested that EGCG treatment ameliorated MI both in vitro and in vivo. More interestingly, EGCG treatment exhibited a pronounced capacity to promote the viability of cardiomyocytes, concurrently suppressing the occurrence of pyroptotic events. It is crucial to underscore that pyroptosis represents a unique mode of cellular demise diverging from apoptosis, stemming from the activation of inflammasomes [22]. Surprisingly, EGCG downregulated MEG3 in OGD/R-treated cells, which might be a key factor to explore the pathway related to the role of EGCG in alleviating MI.

MEG3 is a well-preserved long non-coding RNA (lncRNA) known for its anti-cancer properties. Its expression is commonly reduced in various cancer types due to promoter hypermethylation [23–25]. In the context of MI, MEG3 has exhibited protective effects against myocardial injuries. For example, knockdown of MEG3 was shown to protect H9c2 cells from hypoxia-induced injury through miRNA-183 mediated suppression of p27 [26]. It has been identified as a new target for the prevention of extracellular matrix remodeling in heart diseases. Its downregulation has been linked to the prevention of cardiac fibrosis and diastolic dysfunction [27]. The preventive inhibition of MEG3 reduced cardiac fibrosis and improved diastolic function through impeding MMP-2 induction [10]. Besides, MEG3 knockdown was demonstrated to inhibit pyroptosis and inflammation in OGD/R-treated neurocytes [28]. In this work, we delved into the specific roles of MEG3 in MI. Our findings revealed that a synergistic effect occurred when EGCG treatment is combined with MEG3 knockdown, leading to enhanced cardiomyocyte activity and the inhibition of pyroptosis mediated by AIM2 inflammasome. This observation suggested a potential relationship between the upregulation of MEG3 and AIM2 in the context of MI. To further explore this, we investigated the downstream regulatory targets of MEG3 and identified that TAF15 acted as a RBP of MEG3.

The binding relationship between lncRNAs and RBPs contributed to enhance the stability of downstream mRNAs. TAF15 has been reported function as an RBP that interacts with several lncRNAs in various diseases, such as breast cancer [15], lung squamous cell carcinoma [29], and hepatic ischemia–reperfusion injury [30]. Herein, we demonstrated that TAF15 was indeed the targeted protein of MEG3. Notably, TAF15 overexpression partially reversed the positive effects of EGCG treatment and MEG3 knockdown on cardiomyocytes subjected to OGD/R by upregulating AIM2. AIM2 plays a crucial role in inflammation and pyroptosis by forming the AIM2 inflammasome, which cleaves GSDMD as well as pro-inflammatory cytokines such as IL-1β and IL-18 [31–33]. Notably, the upregulation of AIM2 has been indicated to be closely related to cardiomyocyte death and MI development [14, 34]. Our findings suggested that MEG3 promoted AIM2 expression by binding to TAF15, thereby activating AIM2 inflammasome-mediated pyroptosis. Conversely, EGCG treatment restored cardiomyocyte viability by reducing MEG3 levels. To our best knowledge, this study unveiled, for the first time, the role of MEG3/TAF15/AIM2 in MI development. The data from in vivo experiments also indicated that the synergistic effect of EGCG treatment and MEG3 knockdown contributed to decrease AIM2 expression, while promoting cardiomyocyte viability. This establishes the mechanism by which EGCG alleviates MI through the regulation of the MEG3/TAF15/AIM2 pathway, even in an in vivo context.

Taken together, we elucidated that EGCG protects cardiomocyte from hypoxia-induced pyroptosis by mediating AIM2 inflammasome via the MEG3/TAF15 axis. This work might lay the foundation for further in-depth mechanistic studies and highlight the potential of EGCG as a novel therapeutic agent for the treatment and prevention of MI.

### Supplementary Information

**Additional file 1: Figure S1. (A-F).** The knockdown efficiency of shTAF15, shFUS, shEIF4A3, shDGCR8, shEWSR1, and shELVAL1 was measured by Western blot. **(G).** The overexpression efficiency of oe-MEG3 was detected by qRT-PCR. The results were represented for three individual experiments. *p < 0.05, **p < 0.01, ***p < 0.001.

## Data Availability

Data sharing not applicable to this article as no datasets were generated or analysed during the current study.
