# Supplementary figures and images for: Epigallocatechin gallate prevents cardiomyocytes from pyroptosis through lncRNA MEG3/TAF15/AIM2 axis in myocardial infarction
Source: Chin Med. 2023 Dec 6;18:160. doi: 10.1186/s13020-023-00856-z (PMC10698898; doi:10.1186/s13020-023-00856-z)

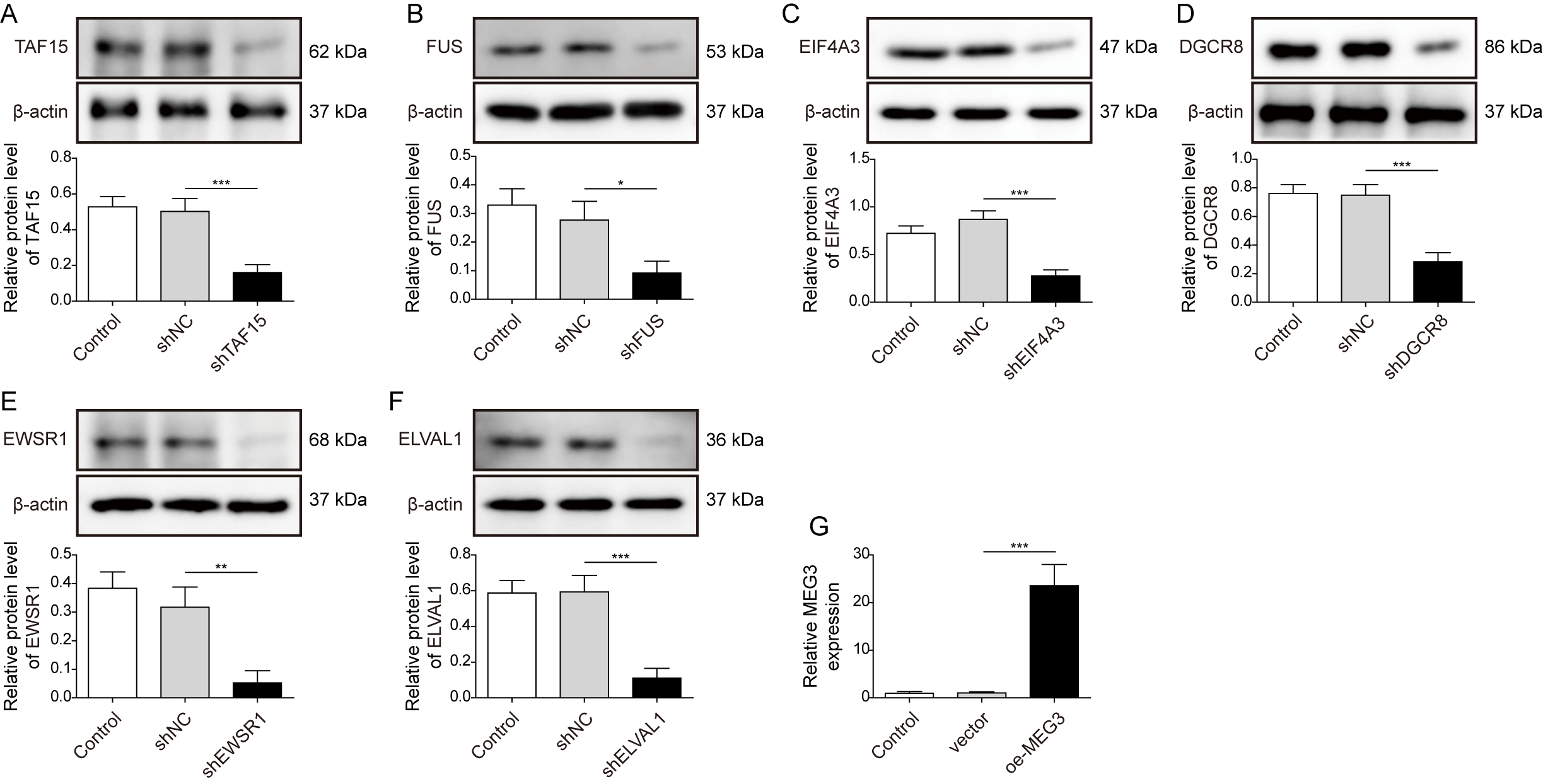

Supplement: Supplementary file 1 — Additional file 1: Figure S1. (A-F). The knockdown efficiency of shTAF15, shFUS, shEIF4A3, shDGCR8, shEWSR1, and shELVAL1 was measured by Western blot. (G). The overexpression efficiency of oe-MEG3 was detected by qRT-PCR. The results were represented for three individual experiments. *p < 0.05, **p < 0.01, ***p < 0.001. [file 13020_2023_856_MOESM1_ESM.tif]
